# Analysis of the Components of a Cognitive-Behavioral Intervention for the prevention of Depression Administered via Conference Call to Nonprofessional Caregivers: A Randomized Controlled Trial

**DOI:** 10.3390/ijerph17062067

**Published:** 2020-03-20

**Authors:** Fernando L. Vázquez, Lara López, Ángela J. Torres, Patricia Otero, Vanessa Blanco, Olga Díaz, Mario Páramo

**Affiliations:** 1 Department of Clinical Psychology and Psychobiology, University of Santiago de Compostela, 15705 Santiago de Compostela, Spain; l.ares@usc.es (L.L.); olga.diaz.fernandez@usc.es (O.D.); 2 Department of Psychiatry, Radiology and Public Health, University of Santiago de Compostela, 15705 Santiago de Compostela, Spainmario.paramo@usc.es (M.P.); 3 Department of Psychology, University of A Coruña, 15001 A Coruña, Spain; patricia.otero.otero@udc.es; 4 Department of Evolutionary and Educational Psychology, University of Santiago de Compostela, 15705 Santiago de Compostela, Spain; vanessa.blanco@usc.es

**Keywords:** depression, nonprofessional caregiver, prevention, cognitive, behavioral, telephone, dismantling

## Abstract

Effective and accessible interventions for indicated prevention of depression are necessary and lacking, especially for informal caregivers. Although telephone-based interventions could increase the accessibility for caregivers, randomized controlled trials are scarce, with no examination of prevention to date. Moreover, the efficacy of specific therapeutic components in preventive cognitive-behavioral programs is unknown. The main objective of this study was to evaluate the efficacy of a telephone-administered psychological preventive intervention in informal caregivers with high depressive symptoms. A total of 219 caregivers were randomized to a cognitive-behavioral conference call intervention (CBCC, *n* = 69), a behavioral-activation conference call intervention (BACC, *n* = 70), or a usual care control group (CG, *n* = 80). Both interventions consisted of five 90-minute group sessions. At the post-intervention, incidence of depression was lower in CBCC and BACC compared to CG (1.5% and 1.4% vs. 8.8%). Relative risk was 0.17 for the CBCC and 0.16 for the BACC, and the number-needed-to-treat was 14 in both groups. Depressive symptoms were significantly lower in BACC and BACC groups compared to CG (*d* = 1.16 and 1.29), with no significant differences between CBCC and BACC groups. The conference call intervention was effective in preventing depression and the behavioral-activation component (BACC) was comparable to the CBCC intervention.

## 1. Introduction

Depression is a mental disorder characterized by a depressed mood and loss of interest in activities once enjoyed [1]. It also presents with many other emotional, cognitive, behavioral, and physiological symptoms that maintain a mutually interactive connection [2]. Due to the clinical and etiological heterogeneity of major depressive disorder, it has been difficult to elucidate its pathophysiology. Current neurobiological theories with the most valid empirical foundation include psychosocial stress and stress hormones, neurotransmitters such as serotonin, norepinephrine, dopamine, glutamate, and gamma-aminobutyric acid (GABA), neurocircuitry, neurotrophic factors, and circadian rhythms [3]. Because all theories of depression apply to only some cases of depressed patients but not others, interventions aimed to address depression, including cognitive-behavioral interventions, should be tailored for the profile of specific patients, and researchers are called to develop novel therapies and formats. Depression affects 322 million people worldwide [4] and is the third-leading cause of years lived with a disability [5]. In 2010 alone it generated an estimated economic cost of 210.5 billion dollars when direct and indirect costs are included [6]. In addition, the disorder is recurrent and chronic [7,8,9]. Between 27% and 42% of people who develop a major depressive episode will suffer further episodes in the next 20 years (see, e.g., [7,8]), and between 12% and 16% of cases will not recover and thus wind up suffering a chronic Major Depressive Disorder (see, e.g., [7,9]). Despite the burden of the disorder and the fact that there are effective pharmacological and psychological interventions, 56.3% of people with depression do not receive treatment [10], and among those who do receive care, only 41% receive adequate treatment [11].

In this scenario, preventive interventions have emerged as one of the most promising alternatives to reduce the prevalence and burden of the disorder, since they reduce the occurrence of new episodes by approximately 21% compared to control conditions [12]. Within the preventive spectrum, prioritized attention should be given to preventive interventions that focus on treating subclinical depressive symptoms to prevent progression to full-blown depressive episodes (i.e., indicated prevention). These interventions are especially cost-effective because they focus on populations with a higher incidence of the disorder because subclinical depressive symptomatology is a factor that doubles the probability of developing major depression [13]. In addition, such preventive interventions are clinically useful in and of themselves because subclinical symptoms are one of the main risk factors for a depressive episode [14], produce clinically significant discomfort and functional impairment [15], have economic costs comparable to those of major depression [16], and increase the risk of mortality [17,18].

Non-professional caregivers are one of the populations in which the prevalence of subclinical and clinical depression is particularly high [19,20,21]. Non-professional caregivers take care of other people in a situation of dependency in their immediate environment to whom they have emotional ties. They do not have an employment relationship or receive financial compensation, although they perform a wide range of tasks that generally take up much of their time [22]. Specifically, it was found that 8.9% of this type of caregivers meet the criteria for a depressive episode [19], and between 22.8% and 46.7% have subclinical depressive symptoms [20,21].

Although these figures support the need to develop depression prevention programs aimed at caregivers, to the best of our knowledge, there are only two randomized controlled trials that have evaluated the efficacy of indicated prevention in this population. In the first, Vázquez et al. (2013) [23] evaluated the efficacy of a brief problem-solving intervention administered in a face-to-face format compared to a usual care control group, finding a lower incidence of depression in the post-intervention group (4.5% vs. 13.1%, *p* = 0.04) as well as lower depressive symptomatology (*M* = 10.7 vs. *M* = 21.2, *Cohen’s d* = 1.54, 95% *CI* 1.20–1.88). In the second, Vázquez et al. (2014) [24] evaluated the efficacy of a cognitive-behavioral intervention administered in a face-to-face format compared to a usual care control group, finding a lower incidence of depression post-intervention in the experimental condition (1.1% vs. 12.2%, *p* = 0.004) as well as lower depressive symptomatology (*M* = 12.4 vs. *M* = 21.3, *Cohen’s d* = 1.05, 95% *CI* 0.73–1.38). Although the results of both trials are promising, the face-to-face administration format limits the reach of both interventions. Many caregivers face a series of barriers that make it difficult to attend face-to-face interventions, including a lack of time, not having another person to replace them while attending sessions, concern for the person cared for during their absence, and obstacles common to other populations such as lack of services (especially in rural areas), transportation issues, the cost of travel, and stigmatization [25].

New technologies can help overcome these obstacles by bringing programs to the homes of those caregivers who cannot travel to receive the psychological help they need. In this sense, the telephone is a device that caregivers have access to and is easy to use, and which allows them to receive the intervention in their homes in a context that provides greater privacy. In addition, there is evidence that psychological interventions for depression over the telephone are at least as effective as those administered in person. A recent meta-analysis [26] found that psychotherapeutic programs for depression administered over the telephone produced a significant reduction in depressive symptomatology compared to control conditions (standardized mean difference [*SMD*] = −0.85, 95% *CI −*1.56 to −0.15), without significant differences with respect to other active conditions, including face-to-face psychotherapy (*SMD* = −0.18, 95% *CI* −0.45, 0.09). In addition, adherence was found to be high, with an average percentage of completed sessions of 73% [26]. 

Although there are psychological interventions administered by telephone for the caregiver population (see, e.g., [27,28]), there are only two randomized controlled trials evaluating the effect of a psychotherapeutic intervention on depressive symptomatology versus a non-active control group [29,30]. Specifically, Pfeiffer et al. [29] found a significant reduction in post-intervention depressive symptoms among the group that received a problem-solving training program (18 calls over 12 months) compared to an information-only control group (*M* = 15.3 vs. *M* = 18.1, *d* = −0.37, 95% *CI* −0.72, −0.01). Similarly, Wilz et al. [30] found a significant reduction in depressive symptomatology after a cognitive-behavioral program for caregivers (12 telephone sessions over six months) compared to a usual care control group (*M* = 18.9 vs. *M* = 20.9, *Cohen’s d* = 0.26). However, none of these trials were specifically aimed at depression prevention. In addition, no cut-off point was established as an inclusion criterion in relation to depressive symptomatology to ensure a homogeneous sample and avoid ceiling and floor effects. Moreover, the individual format and high number of sessions (between 12 and 18) limited the clinical utility of these interventions (with respect to the feasibility of their implementation in real clinical contexts), as well as their efficiency (cost/benefit ratio).

Furthermore, the contribution of the cognitive and behavioral components to therapeutic change in the interventions for indicated prevention of depression is still unknown. Cognitive treatments emphasize the importance of the cognitive change for improvement in depression, although almost all of them include both behavioral and cognitive strategies. In this regard, dismantling designs would make it possible to assess whether the behavioral component of the dismantled protocol is sufficient to reach a level of therapeutic change comparable to the complete package consisting of both cognitive and behavioral strategies, and thus achieve a more focused and parsimonious treatment [31]. Although multicomponent cognitive-behavioral packages are most commonly used for the treatment of depression, two classic trials [32,33] found that behavioral-activation interventions showed an efficacy similar to that of cognitive therapy or a multicomponent cognitive-behavioral intervention. Therefore, it seems reasonable to expect similar results in indicated prevention programs.

The main objective of this study was to adapt and evaluate a group telephone format based on brief cognitive-behavioral intervention that had already been proven effective when administrated to caregivers with high levels of depressive symptoms using a face-to-face format. The study also sought to determine the efficacy of the behavioral-activation component alone compared to the efficacy of the program with all its components (behavioral and cognitive). As a central hypothesis, both interventions were expected to significantly reduce the incidence of depressive episodes and depressive symptoms compared to the control group at the post-treatment.

## 2. Materials and Methods

### 2.1. Design

A randomized controlled clinician trial was conducted (Trial Registration: NCT02292394) using a dismantling design that analyzed the components of a treatment package by eliminating one of the therapeutic components in one of the comparison groups [34]. Specifically, three groups were compared: (a) a cognitive-behavioral conference call intervention (CBCC) using cognitive and behavioral techniques; (b) a behavioral-activation conference call intervention (BACC) using only behavioral techniques aimed at the activation of behaviors; and (c) a control group (CG) in which caregivers received usual care. Participants were randomized after baseline evaluation. An independent investigator (blinded allocation) made random allocation cards using computer-generated random numbers. The researcher kept the original randomization sequences in an inaccessible third place and worked using a copy. The randomization sequence was communicated to the researchers by means of sealed numbered envelopes, one for each participant, with instructions to use them in numerical order. All study participants were evaluated by trained interviewers who were not members of the research team, both before and after the intervention. The evaluators were blinded as to the group to which each participant had been assigned.

### 2.2. Participants

The study participants were recruited through the official registry of non-professional caregivers of the Autonomous Community of Galicia, a region of northwestern Spain with a population of 2,730,337 residents. Participants had to be primary caregivers for a person in a situation of dependency due to various pathologies (officially recognized as such by a public institution) and they had to have a telephone. They also had to score equal to or greater than 16 on the Center for Epidemiological Studies Depression Scale (CES-D) [35,36], have no history of major depression, fail to meet the diagnostic criteria for a major depressive episode, and commit to participate in the evaluations. Exclusion criteria were as follows: (a) psychiatric or psychological treatment in the previous two months that could interfere with the intervention; (b) presentation of other disorders that could act as a confounding variables (e.g., dysthymia, bipolar disorders I and II, cyclothymia, anorexia, psychotic disorders, alcohol or other substance dependence, panic disorder, obsessive compulsive disorder, somatization disorder, hypochondria, or undifferentiated somatoform disorder); (c) a psychological or medical condition that required immediate intervention (e.g., suicidal ideation); (d) significant disease or disorder that made it impossible to conduct the study (e.g., mental deficiency, significant cognitive impairment, severe hearing impairment); (e) participation in another study; (f) an anticipated change of address or institutionalization of the care recipient; and (g) a serious or terminal prognosis of their relative indicated by their care recipient’s physician. 

Based on previous work [24,37,38], this study was designed to detect an 11% difference in the incidence rates of major depressive episodes between the experimental and control conditions. Assuming an alpha level of 0.05, a power of 80%, and an attrition rate of around 1%–7%, the initial sample size was calculated at 213 caregivers (approximately 71 participants per group).

**Figure 1:**
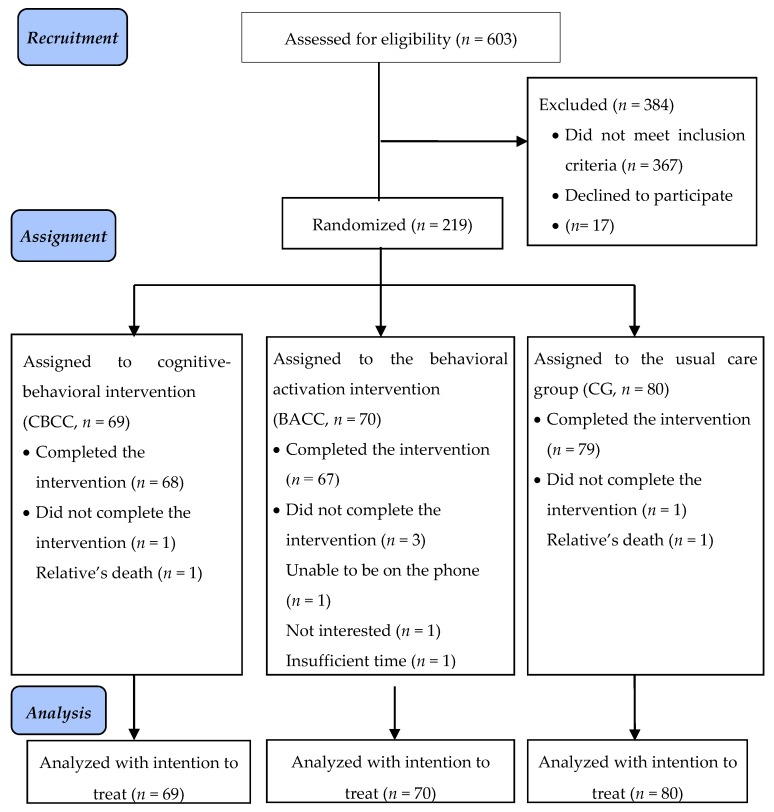
CONSORT 2010 Flowchart.

As shown in Figure 1, a total of 603 caregivers were evaluated, of which 367 did not meet the eligibility criteria. An additional 17 (7.2%) refused to participate. The final sample comprised 219 participants who were randomly assigned to three conditions: 69 participants to CBCC, 70 to BACC, and 80 to CG.

To minimize participant attrition, some of the strategies recommended by Grady et al. (2013) for this type of study were followed [39]. Among others, these strategies included excluding probable losses, making the intervention easy, and holding the sessions at a convenient time. In the end, five people (2.3%) dropped out of the study. One did not complete the cognitive-behavioral intervention, three others in the behavioral-activation group dropped out, and one in the control group dropped out. The reasons given were lack of interest in the intervention, inability to stay on the phone for the duration of the sessions, lack of time, and death of the relative receiving care.

The study respected the principles of the Declaration of Helsinki and was approved by the Bioethics Committee of the University of Santiago de Compostela (Code number 12/09/2012). Caregiver participation was voluntary and no incentives of any kind were given. All participants signed an informed consent.

### 2.3. Instruments

Different instruments were used to evaluate the participants. Specifically, information on the sociodemographic characteristics of caregivers and the care situation was collected using the Questionnaire for Characteristics of the Caregiver and Situation of Care, prepared in a previous study [24,37]. Specifically, information on sex, age, marital status, social class, level of education, main activity of caregivers, sex and age of the care recipient, relationship with the care recipient, diagnosis of the care recipient, time spent caring for the care recipient, and daily hours dedicated to care was collected.

The Structured Clinical Interview from the DSM-5, Clinical Version [SCID-5-CV]) [40], was used to evaluate the presence of a major depressive episode. This is a clinician administered semi-structured interview that covers the most common clinical diagnoses according to the DSM-5. It includes depressive disorder, bipolar disorder, schizophrenia and other psychotic disorders, substance use disorders, anxiety disorders, obsessive-compulsive disorder, post-traumatic stress disorder, attention deficit hyperactivity disorder, and screening for 17 additional disorders. All of the interview modules were administered during the initial evaluation to assess the exclusion criteria, while the post-intervention evaluation used only the module corresponding to depressive disorders. Among its psychometric properties is a Cronbach’s alpha of > 0.80 for all disorders [41].

Symptoms of depression were assessed using the Center for Epidemiological Studies Depression Scale (Center for Epidemiological Studies Depression Scale [CES-D]) [35,36]. This is a 20-item self-reported scale that evaluates the level of depressive symptomatology experienced by the subject during the past week. Each item is scored on a Likert scale consisting of four response options, ranging from 0 (rarely or never) to 3 (most of the time). The total score ranges from 0 to 60, with a higher score reflecting greater symptoms of depression. The average in the general population is 8.7 (*SD* = 8.4), and a score equal to or greater than 16 is considered indicative of a high risk of developing clinical depression. The internal consistency of the Spanish version of the scale (Cronbach’s *α*) was 0.89 [36].

The Customer Satisfaction Questionnaire (CSQ-8]) [42,43] was used to evaluate participant satisfaction with the service received. This questionnaire consists of eight items with four response options, and thus the score ranges from 8 to 32, with higher scores implying greater satisfaction with the service received. The internal consistency of the Spanish version of the scale (Cronbach’s *α*) was 0.80 [43].

Finally, in order to assess the acceptability of the interventions, data on dropouts, attendance at sessions, and completion of homework assignments were collected through an ad hoc register prepared for this study.

### 2.4. Intervention and Control Groups

Prior to the start of the study, cognitive-behavioral conference call intervention (CBCC) and behavioral-activation conference call intervention (BACC) were protocolized and manualized. Subsequently, a pilot study was conducted to evaluate the feasibility of the programs [38]. A previous study outlines the contents of both programs in detail [38].

The cognitive-behavioral conference call intervention (CBCC) was adapted from the indicated preventive intervention for depression based on the multifactor model of Lewinsohn et al. (1985) [2], which has demonstrated its efficacy in the short- and long-term when administered in person in a group format [24,37] using a conference call system. Session 1 explained the concept of depression and the need for active coping with depressive symptoms, and participants were trained in a strategy of activation control (controlled breathing technique), self-reinforcement, and daily mood logging. Session 2 focused on how pleasant activities affect mood, and participants made plans to introduce them into the day-to-day activities. Session 3 addressed how thoughts affect mood and participants were trained in techniques to manage thoughts. Session 4 explained how social contacts affect mood and participants were trained in assertive communication and how to increase social contacts. In Session 5, participants did work during the session aimed at reviewing everything they had learned and preventing relapse.

The adaptations made to the intervention consisted of adjustments related to the change from a face-to-face format to a conference call format; specifically, managing a telephone on-hold system and using skills for telephone support in communication with participants ([44]; e.g., smiling at the beginning of the call, greeting and identifying participants, taking special care to be courteous, speaking slowly, pronouncing clearly, abbreviating the pauses, modulating volume to encourage intonation, increase verbal and paraverbal listening signals, increase the frequency of summaries and recaps), adding a group rule that asked each participant to state their name each time they spoke up during the call, shortening explanations related to program content, and drafting and sending an e-mail or letter to participants written containing material supporting the intervention (i.e., a summary brochure for each session, with the key content and tasks to be performed between sessions).

The behavioral-activation conference call intervention (BACC) was adapted from the cognitive-behavioral conference call intervention for the indicated prevention of depression by Vázquez et al. [24,37], but for the current study, the intervention focused only on the behavioral-activation component. Specifically, Session 1 explained the concept of depression and the need for active coping with depressive symptoms and introduced self-reinforcement and daily mood logging. Session 2 addressed the relationship between mood and pleasant activities and introduced the scheduling of pleasant activities and other behavioral techniques to increase the number of pleasant activities conducted at home. In Session 3, participants continued to work on how to increase pleasant activities, especially outside the home. Session 4 explained how relationships with others influence our mood. Participants were trained in assertive communication and planned how they would increase in social interactions. Session 5 was aimed at reviewing what was learned and preventing relapse. 

CBCC and BACC interventions were applied in a group-format conference call system (approximately five participants per group) over the course of five 90-minute sessions, at the rate of one session per week (one week elapsing between sessions), with an overall duration of five weeks. When a caregiver did not attend a session, their therapist would contact them to provide a summary of the session and provide the homework assignments. The interventions were administered by four psychologists. Prior to the start of the study they received training in administering the interventions: two psychologists in the CBCC intervention and the other two in the BACC intervention. The training consisted of 35 hours of theoretical and practical seminars on the interventions, watching videos, and conducting role-playing exercises with two clinicians. The two clinicians had more than 25 years of experience in the administration of cognitive-behavioral therapies and the use of communication technologies for the implementation of psychological interventions. 

Participants assigned to the usual care control group (CG) did not receive any intervention or material but were not restricted to access to usual care available in their community to treat their depressive symptomatology, including psychological and medical care and social service offerings. A total of 7 out of 80 participants in the CG (8.8%) received pharmacological assistance in their community to treat their depressive symptoms.

### 2.5. Statistical Analysis

The analyses were conducted using the SPSS statistical package (version 24.0) and the freeware program R [45]. The analyses were performed according to the intention-to-treat principle of the Consolidated Standards of Reporting Trials (CONSORT) Statement [46]. This approach assumes that all participants assigned to the groups should be included in the analyses, and thus we used the multiple imputation method to impute the missing values due to sample loss. The lost values were imputed according to the multiple imputation method using the EMB algorithm [47] from the Amelia II program [48]. Fifteen imputations were made. An appropriate imputation model was determined for each of the variables (one that does not exceed 10% of ranges that exclude the straight line y = x) by overimputation. For the combination of parameters of the different models and tests, the *mice* and *miceadds* packages in R were used [49,50].

The incidence of post-intervention depression for the three groups was analyzed. The relative risk (RR) and the necessary number of patients to be treated (NNT) were calculated according to the formulas proposed by Guyatt et al. (1994) [51].

Depressive symptoms among the various conditions and between pre- and post-intervention were compared using linear mixed models (LMM) [52,53]. The Bonferroni correction was applied in the a posteriori contrasts (contrasts between times, contrasts between groups, and contrasts for the time x group interaction). Effect size was calculated taking into account that the estimated models are mixed models, and effect sizes *d* = 0.2 were considered small, *d* = 0.5 as moderate, and *d* = 0.8 as large [54]. The *lme4* package [55] was used to adjust the mixed models and the *emmeans* [56] and *multcomp* [57] packages were used to adjust the *p*-values for the different contrasts and to obtain the effect sizes. 

To determine whether there was a clinically significant change in depressive symptomatology, the formula proposed by Jacobson et al. [58,59,60] was used to find a cut-off point from which to consider a participant as having experienced notable improvement at the clinical level:

$$c=\frac{SD_{0}M_{1}+SD_{1}M_{0}}{SD_{0}+SD_{1}}$$

where:

*SD*_0_ = *SD*_1_ = pretreatment standard deviation (from the experimental and control groups) or standard deviation from the general population; *M*_0_ = mean of the general functional population; *M*_1_ = mean pretreatment (experimental and control groups).

It was estimated that a score below 16.7 at the post-intervention time point indicated a clinically significant change. The percentages for clinically significant change were compared between groups using the Chi-square statistical test.

To analyze the acceptability of the interventions, descriptive statistics were examined, along with dropout frequencies, sessions attended, and intersessional homework tasks performed. To assess satisfaction with the interventions, descriptive statistics were examined using the final score on the CSQ-8. Student’s *t*-tests were used for independent samples for quantitative results to compare these indicators between both interventions, and the Chi-square or Fisher–Freeman–Halton exact test was used for categorical data with an expected frequency of less than five.

## 3. Results

### 3.1. Characteristics of the Sample

Table 1 shows the sociodemographic characteristics and the care situation characteristics for the sample. Participants were mostly women (90.9%), with a mean age of 54.0 years (*SD* = 10.8). Of them, 71.7% had a partner or were married, 52.1% reported belonging to the lower or lower-middle social class, 56.2% had a primary education, and 79.0% had no paid employment.

Regarding the care situation, the majority of care recipients were women (61.2%), with a mean age of 60.8 years (*SD* = 33.1). Among care recipients, 39.3% were the caregiver’s father, followed by 34.2% who were the caregiver’s son. Care recipient diagnosis was dementia in 29.7% of cases, followed by mental disorder, neurological disorder or brain damage in 28.3% of cases. Caregivers had been performing care work for an average of 12.8 years (*SE* = 9.1), averaging 15.8 hours per day (*SD* = 4.1). 

There were no significant differences between CBCC, BACC, and CG for any of the sociodemographic variables or for the pre-intervention care situation.

### 3.2. Incidence of Depression

At the post-intervention time point, 1 of the 69 (1.5%) participants in the CBCC group, 1 of the 70 (1.4%) in the BACC group, and 7 of the 80 (8.8%) in the CG had developed a major depressive episode. When the missing values in the three study groups were replaced as probable cases of depression, the results remained the same. Compared to the CG, the RR for CBCC was 1.45/8.75 = 0.17 (95% *CI* 0.02, 1.31) and the NNT was ≈ 14; the RR for BACC was 1.43/8.75 = 0.16 (95% *CI* 0.02, 1.29) and the NNT was ≈ 14.

### 3.3. Depressive Symptomatology

Table 2 shows the estimated marginal means, standard errors, differences between pre- and post-intervention means, and effect sizes for the CBCC, BACC, and CG groups in depressive symptoms. At the preintervention time point, no statistically significant differences were found in depressive symptomatology among the three groups.

Statistically significant effects were found for the time factor, *F*(5, 2027.05) = 110.607, *p* < 0.001, *η*^2^*p* = 0.434; for the group factor, *F*(2, 11918.94) = 51.175, *p* < 0.001, *η*^2^*p* = 0.092; and for the time x group interaction, *F*(10, 11471.73) = 11.893, *p* < 0.001, *η*^2^*p* = 0.137.

With respect to intragroup changes, the contrasts indicated that there was a significant decrease in depressive symptoms between pre- and post-intervention time points in CBCC, *t* = 11.04, *p* < 0.001, *d* = 1.53, 95% *CI* (1.24–1.83), in BACC, *t* = 12.28, *p* < 0.001, *d* = 1.72, 95% *CI* (1.42, 2.02), and in the *CG*, *t* = 3.82, *p* < 0.001, *d* = 0.49, 95% *CI* (0.24–0.75).

At the post-intervention time point (Table 3), the contrasts in the differences between groups indicate that there were significant differences in depressive symptoms between CBCC and CG, *t* = 6.96, *p* < 0.001, *d* = 1.16, 95% *CI* (0.82–1.50), and between BACC and CG, *t* = 7.72, *p* < 0.001, *d* = 1.29, 95% *CI* (0.95–1.63); there were no significant differences between the two interventions (CBCC and BACC), *t* = −0.76, *p* = 1.000, *d* = −0.13, 95% *CI* (−0.47, 0.21).

### 3.4. Clinically Significant Change

The estimated number of caregivers who achieved a clinically significant change in depressive symptoms at the post-intervention time point was 57 (82.6%) for the CBCC group, 58 (82.9%) for the BACC group, and 31 (38.8%) for the CG group. There were significant differences in clinically significant change between CBCC and GC (χ^2^pooled = 29.7, *p* < 0.001) and between BACC and GC (χ^2^pooled = 31.146, *p* < 0.001), but not between CBCC and BACC (χ^2^pooled = 0.03, *p* = 0.800).

### 3.5. Acceptability and Satisfaction with the Intervention

In relation to dropouts, only five (2.3%) caregivers dropped out of the study; 1 (1.4%) in CBCC, 3 (4.3%) in BACC, and 1 (1.3%) in CG, with no significant differences among the three groups, *p* = 0.49. 

Regarding the number of sessions attended in the CBCC and BACC interventions, it was found that of the five sessions that each intervention included, the average attendance of the participants was 4.4 (*SD* = 1.0) in CBCC and 4.0 (*SD* = 1.3) in BACC; no significant differences were found between these two groups in attendance: *t*(137) = −1.97, *p* = 0.051. A total of 42 (60.9%) of the caregivers in the CBCC group and 33 (47.1%) in the BACC group attended all sessions of the intervention; 58 (84.1%) of CBCC and 52 (74.3%) of BACC attended four or five sessions.

Regarding the performance of the homework assignments, participants of the CBCC group performed an average of 14.9 tasks (*SD* = 4.2) out of the 18 that had been assigned throughout the intervention sessions; while those in the BACC group performed an average of 9.7 (*SD* = 3.3) out of the total of 12 tasks scheduled in this intervention.

Participants in both the CBCC and BACC groups were highly satisfied with the intervention. Caregivers who received CBCC had an average score for satisfaction with the intervention of 28.4 (*SD* = 3.5) and those who received BACC had an average score of 29.1 (*SD* = 2.5). The differences in satisfaction with the intervention between the participants in the two groups were not statistically significant, *t*(123.8) = 1.42, *p* = 0.159.

## 4. Discussion

This study evaluated the efficacy of two telephone-based interventions, a cognitive-behavioral intervention and another intervention that included only behavioral activation strategies, both aimed at the indicated prevention of depression and administered by conference call to caregivers with elevated depressive symptomatology. At the post-intervention time point, it was found that both interventions managed to prevent the occurrence of new cases of major depression and reduced depressive symptoms compared to the control condition, with no significant differences between them. 

The incidence of depressive episodes at the post-intervention time point was 1.5% for CBCC and 1.4% for BACC, which was six times lower than that found in the CG (8.8%). There are no previous randomized controlled trials of telephone-based psychological interventions aimed at caregivers that have assessed the incidence of depression. However, these data are similar to those found at the post-intervention point in a previously performed randomized controlled trial involving a cognitive-behavioral intervention in a face-to-face format [24], which found an incidence of depression of 1.1% in the intervention group and 12.2% in the control group. They are also lower than that found in another study of target depression prevention for caregivers using a face-to-face intervention [23], where the incidence of post-intervention depression was 4.5% in the intervention group and 13.1% in the control group. In comparison with the control group, the RR for the CBCC intervention group was 0.17 and that of the BACC intervention group was 0.16. It was found that either intervention prevented a new case of depression for approximately every 14 caregivers treated. This indicates that both interventions acted as a prevention factor for the appearance of new episodes of depression. These results are consistent with those found in a previous meta-analysis [12], which found an average IRR of 0.74 and an NNT of 13 for the indicated prevention interventions with different populations. These findings are also consistent with the study that used the same cognitive-behavioral intervention administered in a face-to-face format [24], which found an RR of 0.10 and an NNT of 9, as well as the findings for a problem-solving face-to-face intervention [23], which found an RR of 0.34 and an NNT of 12. 

In relation to the effect of the interventions on depressive symptomatology, a significant reduction in depressive symptoms was found for both the CBCC group and the BACC group compared to CG, with large effect sizes (*Cohen’s d* = 1.16 and 1.29, respectively). These findings are consistent with those found in randomized controlled trials evaluating the effect of psychotherapeutic telephone interventions aimed at caregivers with depressive symptoms (e.g., *Cohen’s d* = −0.37 [29], and *Cohen’s d* = 0.26 [30]), although the effect sizes found in our work were considerably higher than in these studies. This superiority may be due to the fact that we selected the sample based on a minimum score for the level of depressive symptomatology and thus avoided ceiling or floor effects. In addition, the absence of significant differences between the two intervention groups indicates that the behavioral-activation component was as effective as the complete cognitive-behavioral intervention in reducing depressive symptoms. In addition, these effect sizes are similar to those found in previous studies of face-to-face interventions for the prevention of depression [23,24], with a *Cohen’s d* of 1.05 and 1.54, respectively, suggesting that the effects of telephone interventions are comparable to those of traditional interventions.

The percentage of participants who experienced clinically significant change in depressive symptoms was significantly higher in both intervention groups (82.6% in CBCC and 82.9% in BACC) compared to the CG (38.8%). However, the rate of clinically significant change in the CG (38.8%) may be due to two contributing factors. First, the mere act of be participating in a study can have an impact in the participants assigned to the CG [34]. In addition, due the nature of the CG (usual care), seven of 80 participants in the CG (8.8%) received pharmacological assistance in their community to treat their depressive symptoms. This relatively high clinically significant change in CG reaffirms the power of the interventions: even when the CG experiences decreases in depressive symptoms, there are still significant differences between the interventions and the CG. Previous research on telephone-based psychological interventions aimed at non-professional caregivers have not assessed clinically significant change. However, these results were higher than the 70.5% post-intervention clinical recovery rate for caregivers receiving the same intervention in a face-to-face format [24] and the 62.5% clinical recovery rate found using more restrictive criteria for a clinically significant change [61]. In addition, the absence of significant differences between the two intervention groups indicates that both interventions were equally effective in the clinically significant change of the participants. These results are very useful at the clinical level, since they reflect the degree to which the intervention impacts the daily life of the recipient, indicating that the majority of caregivers treated with either intervention managed to recover their level of functionality to that of the population without depressive symptoms.

All these findings suggest that the program administered via telephone achieved similar results to those found in other studies in which the programs were administered face-to-face. Therefore, the telephone format maintained its preventive power while improving caregivers’ access to psychological help. In addition, the telephone has the advantage of being a common and affordable device that saves both caregivers and professions from having to travel. Moreover, the conference call format allows several people to be served at the same time. In addition, participants avoid the cost of paying professional caregivers to replace them during their absence to attend services in face-to-face formats. In addition, the absence of differences between the results for the two interventions supports the fact that the behavioral component is a necessary and sufficient ingredient for the prevention of new cases of depression, reduction of depressive symptoms, and clinically significant changes in the caregiver population. These results are consistent with existing evidence on the efficacy of behavioral-activation treatment for depression in adults [62], which is included as an evidence-based treatment by the National Institute for Excellence in Health and Care [63]. Furthermore, it is consistent with evidence showing that behavioral activation is as effective as cognitive-behavioral therapy in the treatment of depression [32,33,64]. This finding is also congruent with the integrative model of depression proposed by Lewinsohn et al. [2] which programs of this study were based on. This model postulates that pathogenic factors of depression (physiological, cognitive, behavioral, emotional) are interconnected; as a result, the treatment of depression could take several different forms. Moreover, although these findings align with preliminary evidence for the treatment of depression [32,33,64], this study is a pioneer, to our knowledge, in analyzing this issue in the field of indicated prevention of depression. 

Furthermore, the percentage of dropouts was low (2.3% in total). The dropout rates are consistent with those for other face-to-face depression prevention programs targeted at caregivers [23,24] and are better than those found in other telephone programs, such as Pfeiffer et al. [29], who reported a dropout rate of 17.2% and Wilz et al. [30], with 13.2%. In addition, the study found high attendance at scheduled sessions (*M* = 4.4 sessions for CBCC and *M* = 4.0 for BACC), similar to those found for other in-person targeted prevention programs [24] and the 73% average attendance found for telephone programs for the treatment of depression [26]. Homework completion was high (*M* = 14.9 of 18 for CBCC and *M* = 9.7 of 12 for BACC). This finding is important, because previous research has found that homework is essential to achieving positive therapeutic results in the caregiver population [65]. The fact of planning simple weekly tasks that are applicable in daily life, and subsequent review and feedback about their achievement, likely contributes to this high compliance rate. In addition, satisfaction was also high (*M* = 28.4 for CBCC and *M* = 29.1 for BACC), a result consistent with the results of a previous review study [66] that found high satisfaction among participants in psychological interventions for depression administered by phone. These results may be due to the fact that the telephone format facilitated access to services, its brevity was in keeping to the limited time available to caregivers, the content was understandable and simple, the staff were very approachable, and the wait times were short. 

This study presents several strengths. It is the first randomized controlled trial to evaluate the efficacy of a brief cognitive-behavioral intervention for indicated depression prevention targeted at caregivers administered through a conference call system using a dismantling strategy to analyze the contribution of the behavioral-activation component to the results arising from the intervention. It is one of the few studies that has been conducted using specific inclusion and exclusion criteria to select a sample with a homogeneous level of depressive symptomatology, based on a clearly explained theoretical model, using a manualized treatment program, trained professionals, and blind evaluation of the results through the use of evaluation instruments with adequate psychometric properties. However, certain limitations should be noted. First, the lack of follow-up prevents us from knowing whether the improvements that occurred following the interventions were maintained over time. In preventive science, it is important to know whether the intervention prevents the occurrence of new cases of depression or merely temporarily delays their onset. Future work must include long-term follow-up. Second, we do not know what specific mechanisms underlie the effects of the components that were studied. The dismantling design allows us to identify critical components of the program but does not detect the possible processes operating behind the changes those components achieved [67]. Third, most of the sample consisted of women, which makes it difficult to generalize the results to male caregivers. However, this disadvantage is minimized when we consider that most people caring for a person in a dependent situation are women [68].

This study has important implications for research and clinical practice. It demonstrates the efficacy of a preventive program administered through telephone conference calls among non-professional caregivers, and it is the first study to analyze the contribution of the behavioral-activation component to a cognitive behavioral multicomponent program for indicated prevention of depression. Moreover, the two telephone-based interventions, CBCC and BACC have a series of characteristics that make their application into routine clinical care viable. First, the telephone format makes it possible for participants to receive psychological care from their homes. This innovative format increases the accessibility of services and provides greater coverage of care, especially in more remote areas and among underserved populations. Second, the short duration of the program (five weeks) and group format makes it time-efficient and possible to complete with only one clinician. Thus, these programs would reduce health care system costs and wait times to receive care. In addition, the efficacy of the behavioral-activation component means that it may be possible to provide an intervention that is more straightforward and easier to administer than programs with multiple components. Finally, the high adherence and satisfaction with the two interventions demonstrates that both programs are acceptable to patients. At a societal level, this study focuses on preserving the mental health of a population whose work is essential for the maintenance of current systems to promote the health and well-being of the population. Finally, this study provides an accessible, innovative, and effective intervention to avoid the occurrence of a disorder whose high prevalence and associated burden constitute an important public health concern.

## 5. Conclusions

This study evaluated the efficacy of a cognitive-behavioral intervention for indicated depression prevention targeted at non-professional caregivers administered through telephone conference calls, both the full cognitive and behavioral components and only with the behavioral-activation component, compared to a control group of usual care. Both interventions managed to reduce the incidence of depression and to reduce levels of depressive symptomatology among the participants after the intervention, with no differences between the two intervention groups. These results support the utility of such programs and their applicability in health care systems. Telephone administration increases accessibility by overcoming many space and time limitations; in addition, their brevity and group format would save costs and reduce waiting lists. Furthermore, the study provides evidence that behavioral activation constitutes a critical component of cognitive-behavioral intervention, which will help in the design of shorter and equally effective programs for indicated depression prevention aimed at the caregiver population.

## Figures and Tables

**Table 1:** Sociodemographic characteristics and the care situation for the sample.

Variables	Total		CBCC		BACC		CG	
	*N* = 219	*%*	*n* = 69	*%*	*n =* 70	*%*	*n* = 80	*%*
**Sex**								
Men	20	9.1	7	10.1	3	4.3	10	12.5
Women	199	90.9	62	89.9	67	95.7	70	87.5
**Age**								
*M*	54.0		54.8		54.5		52.9	
*SD*	10.8		10.7		11.0		10.7	
Range	25–76		34–75		32–74		25–76	
**Marital status**								
Single	25	11.4	7	10.2	9	12.9	9	11.2
Married, lives as a couple	157	71.7	51	73.9	52	74.2	54	67.5
Separated, divorced, widowed	37	16.9	11	15.9	9	12.9	17	21.3
**Social class**								
Low/lower middle	114	52.1	36	52.2	39	55.7	39	48.7
Middle/upper middle	105	47.9	33	47.8	31	44.3	41	51.3
**Level of education**								
Can read and write	27	12.3	5	7.3	12	17.1	10	12.5
Primary	123	56.2	39	56.5	38	54.3	46	57.5
Secondary/University	69	31.5	25	36.2	20	28.6	24	30.0
**Primary activity**								
Active worker	46	21.0	11	15.9	16	22.9	19	23.7
Unemployed/retired	173	79.0	58	84.1	54	77.1	61	76.3
**Care recipient sex**								
Male	85	38.8	28	40.6	28	40.0	29	36.2
Female	134	61.2	41	59.4	42	60.0	51	63.8
**Care recipient age**								
*M*	60.8		59.9		67.6		55.5	
*SD*	33.1		32.7		30.0		35.2	
Range	1–100		1–99		3–98		3–100	
**Relationship**								
Father/mother	86	39.3	27	39.1	32	45.7	27	33.7
Spouse/partner	12	5.5	2	3.0	4	5.7	6	7.5
Son/daughter	75	34.2	27	39.1	17	24.3	31	38.8
Other	46	21.0	13	18.8	17	24.3	16	20.0
**Care recipient diagnosis**								
-Diseases of the skeletomuscular/connective tissue/cardiovascular/respiratory systems	53	24.2	13	18.8	18	25.7	22	27.5
-Chromosomal/congenital/perinatal abnormalities	39	17.8	11	15.9	13	18.6	15	18.8
-Mental/neurological disorders/brain damage	62	28.3	21	30.5	16	22.8	25	31.2
-Dementias	65	29.7	24	34.8	23	32.9	18	22.5
**Care duration (years)**								
*M*	12.8		13.9		12.8		11.9	
*SD*	9.1		9.8		9.0		8.5	
Range	1–47		1–47		2–47		2–46	
**Daily hours of care**								
*M*	15.8		15.3		16.2		15.9	
*SD*	4.1		4.4		3.8		4.0	
Range	1–20		3–20		1–18		5–20	

CBCC: Cognitive-behavioral conference call intervention; BACC: Behavioral-activation conference call intervention; CG: Usual care control group.

**Table 2:** Estimated marginal means, standard errors, and pre-post comparison on depressive symptomatology (CES-D) per group.

Group	PRE	POST	*t*	*SE*	*p*	Effect Size	*95% CI*	
	*M (SE)*	*M (SE)*					Min	Max
CBCC	22.3 (0.86)	11.3 (0.87)	11.044	0.989	<0.001	1.53	1.24	1.83
BACC	22.7 (0.86)	10.4 (0.88)	12.280	0.999	<0.001	1.72	1.42	2.02
CG	23.1 (0.80)	19.6 (0.81)	3.815	0.920	<0.001	0.49	0.24	0.75

CBCC: Cognitive-behavioral conference call intervention; BACC: Behavioral-activation conference call intervention; CG: Usual care control group. 95% CI: 95% confidence intervals; min: Minimum confidence interval; max: Maximum confidence interval.

**Table 3:** Pairwise comparison of intervention effects on post-intervention CES-D.

Comparison	*t*	*SE*	*p*	Effect Size	*95% CI*	
					Min	Max
CBCC vs. CG	6.963	1.188	<0.001	1.16	0.82	1.50
BACC vs. CG	7.724	1.192	<0.001	1.29	0.95	1.63
CBCC vs. BACC	−0.760	1.237	1.000	−0.13	−0.47	0.21

Note. CBCC: Cognitive-behavioral conference call intervention; BACC: Behavioral-activation conference call intervention; CG: Usual care control group; 95% CI: 95% confidence intervals; min: Minimum confidence interval; max: Maximum confidence interval.
